# Electronic Structure of B20 (FeSi-Type) Transition-Metal Monosilicides

**DOI:** 10.3390/ma12172710

**Published:** 2019-08-24

**Authors:** Dmitry A. Pshenay-Severin, Alexander T. Burkov

**Affiliations:** Ioffe Institute, Saint Petersburg 194021, Russia

**Keywords:** electronic structure, monocilicide, B20 crystal structure, topological semimetal

## Abstract

Monosilicides of transition metals crystallizing in a B20 (FeSi-type) structure (space group P2${}_{1}$3, #198) possess a wide range of specific properties. Among them are semiconductors, metals, and paramagnetic, diamagnetic, and ferromagnetic compounds. Some of them were studied as promising thermoelectric materials. Recently, B20 monosilicides have attracted attention as a new class of topological semimetals with topological charge greater than unity. In the present work, we analyze the electronic structures of B20-type monosilicides of the fourth, fifth, and sixth periods of the Periodic Table in order to reveal their common features and peculiarities. To make this analysis more consistent, we performed a density-functional study of the electronic structures of the monosilicides in a unified manner. We reviewed the results of previous calculations and the available experimental data, comparing them with our results. The band structures of ReSi and TcSi not found in the literature were calculated and analyzed as well. The topological properties of these materials and of some isostructural germanides and stannides were investigated. Analysis reveals the current understanding of electronic structures and properties of this compound group.

## 1. Introduction

Monosilicides of transition metals that crystallize in a cubic noncentrosymmetric B20 structure (space group P2${}_{1}$3, #198) have many interesting magnetic, topological, and transport properties. In recent years, interest in the topological properties of solids has increased. Many monosilicides exhibit unusual properties both in real and in reciprocal space. For example, in MnSi and in isostructural MnGe, unusual magnetic properties related to the existence of the skyrmion phase were observed [1,2]. Recently, it was discovered that the semimetallic CoSi and RhSi have nontrivial electronic structure topology [3,4,5,6]. While the low-energy electronic structure in Weyl semimetals contains an even number of doubly-degenerate band-touching points, carrying topological charge ±1, in CoSi and RhSi the degree of degeneracy is higher, and topological charges are greater than unity. The high degree of degeneracy is caused by the symmetry of their crystal lattice [3]. In contrast to Weyl nodes, fermions corresponding to nodes with topological charge greater than unity do not exist in relativistic quantum-field theory because they are prohibited by Lorentz invariance. Thus, in Ref. [3], they are called “new fermions”.

The study of the band structure of CoSi and its alloys with FeSi started with a semimetallic model featuring overlapping parabolic valence and conduction bands [7,8]. More recent band structure calculations in the framework of density functional theory demonstrated that their electronic structure is more complex. Band structure calculations of CoSi were done without taking into account the spin–orbit coupling (SOC) [9,10], as well as including SOC [4,6,11]. The band structure of the isostructural RhSi was studied too [12,13]. Symmetry analysis showed that the electronic structure of crystals with P2${}_{1}$3 space group (#198) contains a sixfold degenerate band-touching node at the *R* point of the Brillouin zone (BZ) [3]. Density functional theory (DFT) calculations showed that the electronic structure of CoSi and RhSi indeed has nonzero topological charge and Fermi arcs, connecting projections of band-touching nodes at Γ and *R* points on the surface Brillouin zone [4,5,6]. The band-touching nodes in bulk electronic spectrum and the Fermi arcs for CoSi were recently experimentally observed in angle-resolved photoemission spectroscopy (ARPES) experiments [14,15,16]. Although the degree of degeneracy is restricted by space-group symmetry, the energy position of these nodes and the peculiarity of the corresponding surface Fermi arcs should be determined by band structure calculations. Thus, it is interesting to compare their band structures and topological properties with other isostructural transition metal monosilicides and other compounds, such as CoGe, RhGe, and RhSn.

The thermoelectric properties and band structure of CoGe within a generalized gradient approximation (GGA) full-potential approach were studied in Ref. [17]. The magnetic and superconducting properties of RhGe and its band structure were investigated in Refs. [18,19] with a GGA plane-wave approach. The band structure of RhSn, taking into account the spin–orbit coupling, has not, to the best of our knowledge, been calculated.

Monosilicides of the eighth group (Fe, Ru, and Os) are semiconductors. According to the authors of [20,21,22], they are topologically trivial insulators. The electronic band structure and dynamical lattice properties of Fe(Ru,Os)Si were calculated in Ref. [23]. The band structure and the elastic properties of RuSi, RuGe, and OsSi were calculated in Ref. [24] by means of the plane-wave pseudopotential method in GGA approximation. Elastic moduli were calculated in Ref. [25] at ambient pressure, and in Ref. [26] under high pressure.

Band gap $\epsilon_{g}$ of FeSi was determined from experimental data on temperature dependences of resistivity and optical conductivity [27,28,29] and it yielded values in the range of 0.054–0.06 eV at temperatures below 200 K; at higher temperatures, conductivity resembled that of a dirty metal.

At temperatures below 1573 K, RuSi crystallizes in a semiconducting FeSi-type structure, while, at higher temperatures, metallic CsCl-type structure is stable [30]. The measurements of resistivity and optical conductivity revealed that, in a semiconducting state at temperatures of about 500 K, the band gap is $\epsilon_{g}\approx$ 0.2–0.3 eV [30]. Around room temperature, the compound demonstrates extrinsic conduction, which is changed into activated behavior at lower temperatures, with activation energy of only about 8 meV. Below 50 K, crossover to hopping-type conduction was observed [30].

Similar results were obtained for RuSi and OsSi based on the study of transport properties [31]. The band gap of RuSi was estimated as 0.26 eV above 400 K, while activation energy at 150 K was 20 meV [31]. The resistivity of OsSi is typical for a degenerate semiconductor, showing intrinsic semiconducting behavior only above 1000 K, thus the band gap was estimated to be larger than 0.26 eV [31]. The magnetic susceptibility of both materials is negative, down to 5 K [31].

MnSi is an itinerant magnet at temperatures below $T_{c}=$ 30 K [32]. Neutron diffraction showed that, in a weak magnetic field *B*, MnSi has a helical magnetic structure with a long period of about 180 Å along the $\left[111\right]$ crystallographic direction [32]. When the magnetic field increases, the magnetic structure first becomes conical, directed along the magnetic field, and then changes to ferromagnetic [2]. Interestingly, the B−T phase diagram near $T_{c}$ in the conical phase, contains small region of the skyrmion phase [2]. $T_{c}$ decreases with pressure, and the magnetic order disappears above the quantum critical point of 1.46 GPa [33,34]. The band structure of MnSi was calculated in Ref. [35] using the self-consistent augmented plane-wave method with Slater’s Xα approximation for exchange interaction.

Trends in the density of states (DOS) in monosilicides of Cr, Mn, Fe, Co, Ru, Rh, and Os with a B20 structure were analyzed in Ref. [36] using the plane-wave pseudopotential method. Ferromagnetic-to-paramagnetic phase transition under pressure in MnSi was studied theoretically using self-consistent linear muffin-tin orbital method within atomic-sphere approximation [37]. Band structure and magnetic property peculiarities were studied using the full-potential method in local density approximation (LDA) [38]. These works agree that the DFT ground state for MnSi is ferromagnetic, but calculations gave values of a magnetic moment equal to 1 $\mu_{B}$, which is considerably larger than the experimental value of 0.4 $\mu_{B}$ [38]. The inclusion of on-site correlation of Mn d-electrons in the framework of the LDA+U approach allowed obtaining an experimental value of the magnetic moment for Hubbard parameter U∼6 eV [39,40]. Due to the large period, description of a helical magnetic structure is difficult within an ab initio approach. Dzyaloshinskii–Moriya interaction appeared to be important in the description of long-period helical magnetic structures in MnSi [41]. Parameters of this interaction were obtained, and its influence on magnetic-phase transition in MnSi was discussed [40,42]. Qualitatively, the band structure calculated in the DFT framework correlated with ARPES data near the Fermi level [43], although more refined self-consistent quasiparticle GW-calculations better describe an experimentally obtained electronic spectrum, away from the Fermi level [43].

The band structures of technetium (Tc) and rhenium (Re) monosilicides were not found in the literature, possibly because Tc is radioactive while, in the case of Re, more attention has been paid to studies of ReSi${}_{1.75}$ (see recent review [44]).

From the sixth group monosilicides, only CrSi crystallizes in a B20 structure. CrSi is paramagnetic metallic material, and its band structure was calculated with extended Huckel theory [45,46], using plane-wave pseudopotential [12] and, recently, using the full-potential augmented plane-wave method [47], both using LDA approximation. LDA+U parameters were calculated for the whole series of Cr(Mn,Fe,Co)Si compounds using constrained DFT calculations [47].

Besides topological and magnetic properties, thermoelectric transport in these materials has widely been studied. The most studied ones are CoSi and its solid solutions with FeSi or NiSi [7,8,10,27,48,49,50,51]. Seebeck coefficient *S* and electrical conductivity σ of the whole series of solid solutions of CrSi–MnSi–FeSi–CoSi–Co${}_{0.85}$Ni${}_{0.15}$Si were studied experimentally and theoretically in Ref. [10]. The largest negative Seebeck coefficient at room temperature of about −80 μV/K was observed in CoSi at stoichiometric composition [7,10,48]. Seebeck coefficient magnitude decreases with the substitution of Co with Ni and with Fe. CoSi alloys with large FeSi contents have a positive Seebeck coefficient. In Co${}_{1-x}$Fe${}_{x}$Si alloys, the Seebeck coefficient reaches a maximum of 60 μV/K at about x≈0.25, and then decreases and again changes its sign to negative when *x* approaches unity [10].

Power factor $S^{2}\sigma$ in CoSi is rather high; it can reach values of about 5.5 mW/mK${}^{2}$ at 350–400 K, which is larger than the power factor of Bi${}_{2}$Te${}_{3}$ (4 mW/mK${}^{2}$). However, due to large lattice thermal conductivity (about 10 W/mK at room temperature), its thermoelectric efficiency is comparatively low (ZT= 0.15–0.2 at 400–600 K) [7]. Similar values were obtained in Ref. [17] for CoGe and its solid solutions with FeGe and NiGe. The largest Seebeck coefficient (−82μV/K) was observed in pure CoGe. The maximum value of the thermoelectric figure of merit parameter ZT was as high as 0.11 at 300 K. A similar value (ZT=0.11 at 350 K) was observed in CoSi${}_{1-x}$Ge${}_{x}$ with 10 at.% of Ge [52]. A large power factor of about 6 mW/mK${}^{2}$ was reported for CoSi${}_{0.95}$Ge${}_{0.05}$ at room temperature. Even though Ge is isoelectronic to Si, both conductivity and the Seebeck coefficient increase in these alloys with Ge content. This behavior was attributed to changes in band structure and DOS [53]. However, reduction of lattice thermal conductivity in the alloys was partially compensated by the large electronic thermal conductivity. Therefore, no essential improvement of ZT in the CoSi${}_{1-x}$Ge${}_{x}$ alloys has been found. Other attempts to improve the thermoelectric efficiency of CoSi by alloying (doping) and by nanocrystallization have not been successful [44,54,55,56,57].

In FeSi, maximum Seebeck coefficient strongly depends on doping. Without doping, it can reach 1200 μV/K in single crystal, and 500 μV/K in polycrystalline samples [58]. The temperature of these maxima is about 50 K. When doped with cobalt, the Seebeck coefficient becomes negative (−50μV/K) [58] with the maximum at the same temperature of 50 K. Doping by 4at.% of Ir leads to S≈−130μV/K [58] at about 75 K. Strong lattice thermal conductivity reduction due to the large mass difference of Fe and Ir allowed to obtain ZT=0.125 at 100 K in FeSi [58].

RuGe, RuSi${}_{0.5}$Ge${}_{0.5}$, and RuSi also demonstrated typical for semiconductors temperature dependence of the Seebeck coefficient with maxima of 100 μV/K (175 K), 300 μV/K (200 K) and 300 μV/K (300 K), respectively [31]. Maxima temperatures correlate with the increase of the band gap in this series of compounds, from 0.15 eV in GeSi to 0.2 eV in RuSi${}_{0.5}$Ge${}_{0.5}$, and 0.26 eV in RuSi [31]. OsSi and alloy Ru${}_{0.5}$Os${}_{0.5}$Si have large hole concentrations, presumably due to deviation from stoichiometry, and demonstrate a small Seebeck coefficient (50 and 100 μV/K, correspondingly), linearly increasing up to 500 K [31]. The onset of intrinsic conduction was observed only close to the upper limit of measurements, and band gap was estimated to be greater than 0.26 eV. Thermoelectric efficiency ZT in these materials does not exceed 0.02–0.025.

In metallic MnSi and CrSi, the Seebeck coefficient is quite small. It is positive (about 30 μV/K) for MnSi and negative in CrSi (about −15μV/K) [10].

Despite a great number of calculations of the electronic structures of these compounds, they were made using different approximations. Thus, it is useful for their overview and comparison to be based on a unified approach for the whole range of monosilicides, and to add the band structures of TcSi, ReSi, and RhSn that were not considered before. Due to emerging attention to their band structure topology and persistent interest to their thermoelectric applications, here we also discuss the influence of the electronic structure on these properties.

## 2. Calculation Methods

Band structure calculations were performed in the framework of density functional theory in Perdew–Burke–Ernzerhof (PBE) generalized gradient approximation [59] as implemented in VASP [60,61]. SOC was taken into account in all calculations, but for comparison, band gaps were also obtained without SOC. Total energy convergence was better than 1 meV/at for the plane-wave cutoff, varying in the range of 350–500 eV. For consistency, we used the value of 500 eV and an 8 × 8 × 8 Γ-centered Monkhorst–Pack grid for all compounds throughout the calculations with the exception of hybrid functional calculations. In the latter case, we used a cutoff of 350 eV for FeSi, 400 eV for RuSi, and 450 eV for OsSi. Gaussian smearing with a smearing width of 0.026 eV was used. Using these parameters, equilibrium lattice constants were obtained by fitting to the Birch–Murnagan equation of state, allowing for the relaxation of atoms in the unit cell until residual forces were less than 1 meV/Å. The density of states was obtained in VASP on a 32 × 32 × 32 *k*-points grid using tetrahedron integration.

For a more detailed study of the band structure, transport and topological properties, a tight-binding Hamiltonian was obtained using the Wannier90 software package [62], which includes the highest s- and d-states of transition metals and s- and p-states of silicon. Wannier interpolation [62] was used for plotting constant energy surfaces on a 50 × 50 × 50 *k*-point grid. The visualization of Fermi surfaces and crystal structure was made in XCrySDen [63]. Using a tight-binding Hamiltonian, topological properties were calculated with the help of the Wannier Tools package [64]. The Seebeck coefficient was calculated in BoltzWann program [62] in constant relaxation time approximation, using Wannier interpolation on 192 × 192 × 192 *k*-point grid.

## 3. Results of Band Structure Calculations

### 3.1. Crystal Structure Parameters

The monosilicides that we study here were selected based on literature data and the Materials Project database [65]. The following transition-metal monosilicides with FeSi-type (B20) structure (space group P2${}_{1}$3, #198) were found [65]: CrSi, MnSi, FeSi, CoSi, NiSi, TcSi, RuSi, RhSi, ReSi. In addition, CoGe, CoSn, RhGe, and RhSn were considered to compare their topological properties with those of CoSi. Most of the compounds have a stable FeSi-type structure, although density functional calculations predicted that the most stable phase differs from B20 for CrSi, NiSi, RhSi, OsSi, IrSi, CoGe, CoSn and RhGe. However, the samples of CoGe and RhGe with a B20 structure were obtained using high-pressure synthesis [2,66]. Formation and decomposition energies for these compounds are given in Table 1 on the basis of data from Ref. [65].

**Figure 1:**
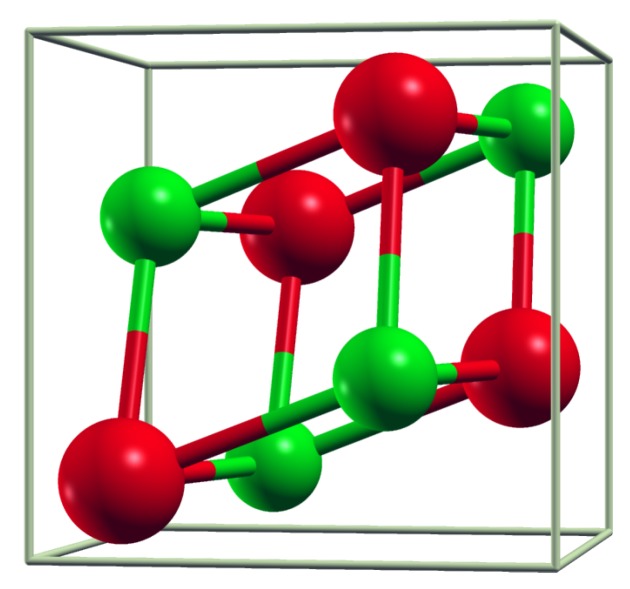
Unit cell of B20 (FeSi-type) crystal structure. Red and green balls are metal and Si atoms, respectively.

The unit cell in FeSi-type materials is cubic but lacks inversion symmetry. There are four metal and four silicon atoms in the unit cell, as shown in Figure 1. Atomic positions in the crystalline coordinates are (x,x,x), (−x+1/2,−x,x+1/2), (−x,x+1/2,−x+1/2), and (x+1/2,−x+1/2,−x) with different *x* for metal ($x_{\mathrm{Me}}$) and silicon ($x_{\mathrm{Si}}$). It can also be described as a cubic supercell of NaCl structure with four formula units, but with atomic positions stretched in the [111] direction, which leads to a deviation of $x_{\mathrm{Me}(\mathrm{Si})}$ from values in the rock-salt structure ($x_{\mathrm{Me}}$ = 0.25, $x_{\mathrm{Si}}$ = 0.75) [67].

Calculated equilibrium lattice parameters and bulk moduli are given in Table 2 in comparison with the experimental values. Qualitatively, the dependence of the lattice constants on the transition metal was reproduced by calculations. Lattice constant $a_{0}$ decreases with the increase of transition-metal number inside the same row of the Periodic Table, but becomes larger while passing from the fourth to the fifth and sixth rows. This behavior correlates with the atomic radii of metal atoms. Interestingly, while, for the fourth-row transition-metal monosilicides, the calculated values of lattice constants are smaller than the experimental ones, for the fifth- and sixth-row monosilicides, the theoretical lattice parameters are larger than the experimental ones. The maximum deviation of theoretical $a_{0}$ was found for MnSi (1.3%) and OsSi (1.35%) while, for other monosilicides, deviation was less than one percent.

CrSi and MnSi have a ferromagnetic ground state with theoretical magnetic moments of 0.48 and 1 $\mu_{B}$ per transition-metal atom. Fitting a state equation for the case of a nonzero magnetic moment gives larger $a_{0}$ values and reduces deviation from experimental values for both CrSi (0.8%) and MnSi (0.9%). For other considered monosilicides, DFT predicted a nonmagnetic ground state.

The experimental values of the bulk moduli ($B_{0}$) increase inside the fourth row from MnSi to CoSi [68]. DFT calculations cannot grasp this trend. For MnSi, FeSi, and CoSi, the calculated moduli were almost the same. Only a marginal decrease of $B_{0}$ was obtained for MnSi when allowing for a magnetic ground state. In general, the theoretical overestimation of the bulk moduli for MnSi, FeSi, and CoSi correlates with the underestimation of the lattice constants compared to the experimental values. The best agreement was found for CoSi, for which error in $a_{0}$ was 0.3%, and in $B_{0}$ was 6%.

### 3.2. Band Structure and Topological Properties of Semimetallic Co and Rh Monosilicides and Monogermanides, and of RhSn

**Figure 2:**
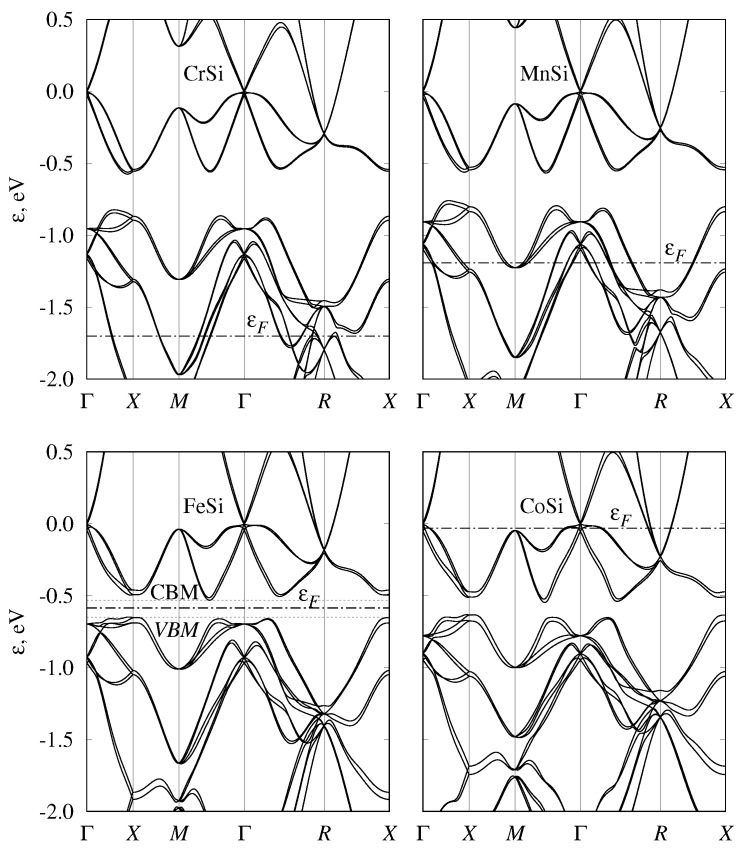
Band structure of CrSi, MnSi, FeSi, and CoSi with B20 (P2${}_{1}$3, #198) crystal structure. Energy is measured relative to fourfold band crossing at Γ point. Dash-dotted line shows Fermi level position. Dotted lines on FeSi panel indicate the valence band maximum (VBM) and conduction band minimum (CBM) energies along high-symmetry directions.

**Figure 3:**
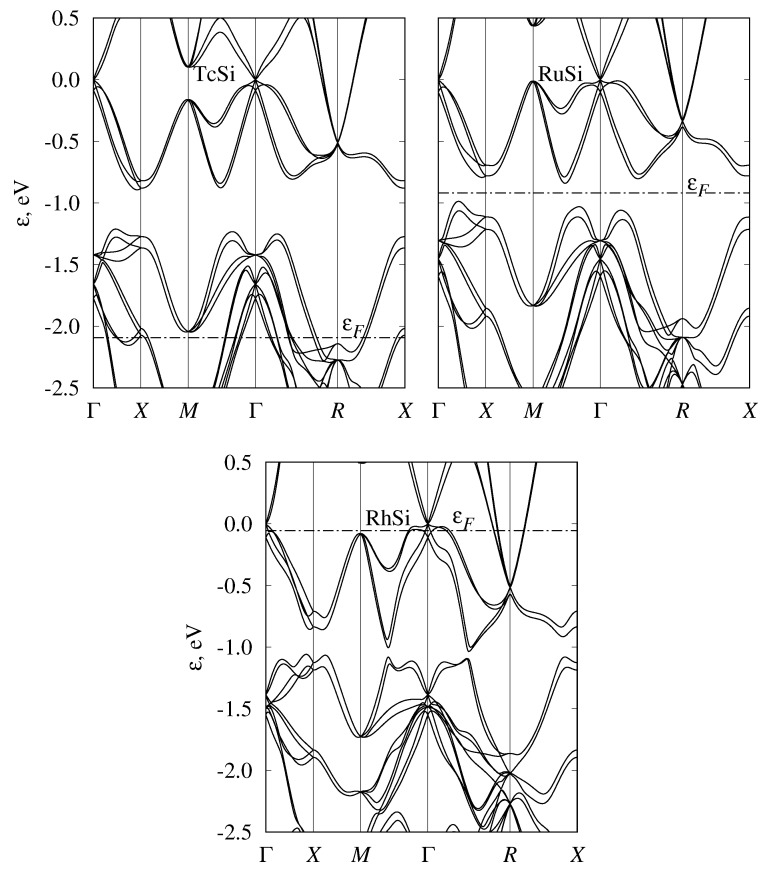
Band structure of TcSi, RuSi, and RhSi with B20 (P2${}_{1}$3, #198) crystal structure. Energy measured relative to fourfold band crossing at Γ point. Dash-dotted line shows Fermi level position.

**Figure 4:**
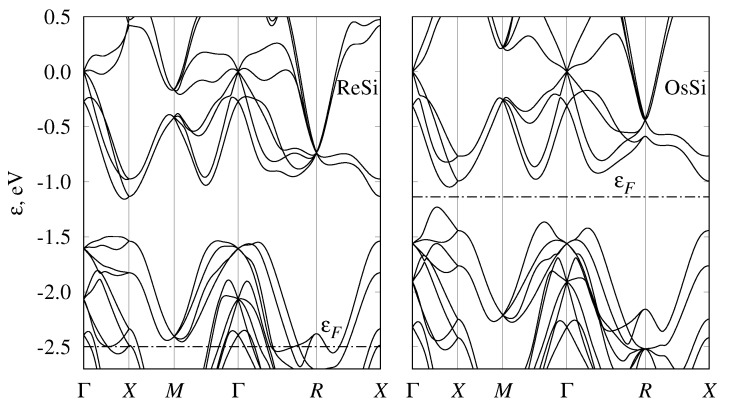
Band structure of ReSi and OsSi with B20 (P2${}_{1}$3, #198) crystal structure. Energy measured relative to fourfold band crossing at Γ point. Dash-dotted line shows Fermi level.

**Figure 5:**
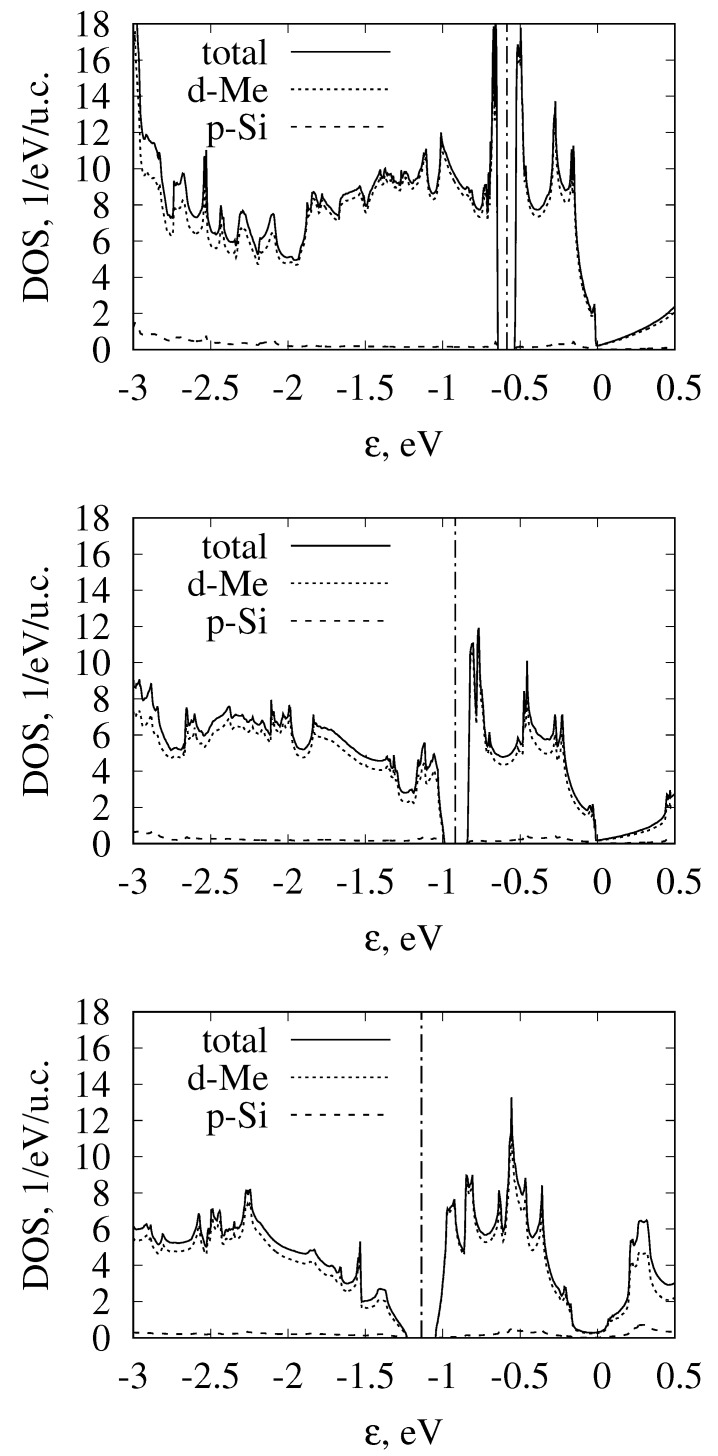
Total and projected density of states in FeSi, RuSi, and OsSi. Fermi level is shown by vertical dash-dotted line.

While we started the discussion from semimetallic monosilicides, we note that the band structures of all considered transition-metal monosilicides of the fourth, fifth, and sixth periods are given in Figure 2, Figure 3 and Figure 4 respectively. To emphasize the similarities of the electronic structures, energy was measured relative to the fourfold band crossing at the Γ point. Figure 5 shows the density of states for the three selected semiconducting compounds (FeSi, RuSi, and OsSi) representing the monosilicides of fourth, fifth, and sixth rows of the Periodic Table. In the whole considered energy range, the main contribution to DOS came from metal d-states and silicon p-states.

**Figure 6:**
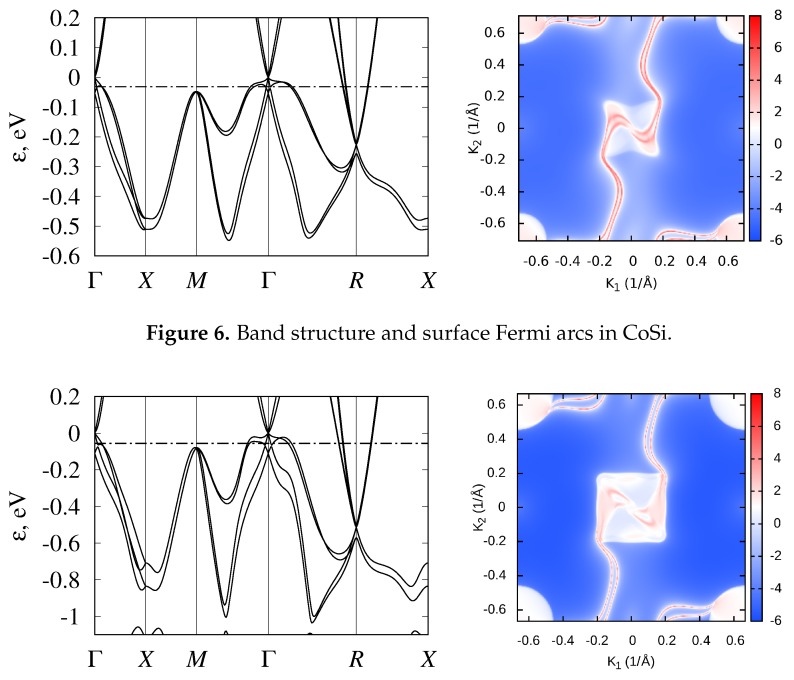
Band structure and surface Fermi arcs in CoSi.

**Figure 7:**
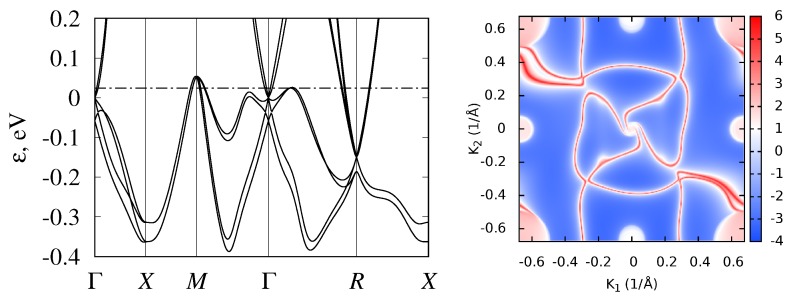
Band structure and surface Fermi arcs in RhSi.

In CoSi and RhSi, Fermi energy is located just below the fourfold band crossing at Γ point (ϵ=0) near the DOS minimum. Thus, they demonstrate semimetallic behavior. This band crossing, together with the sixfold band crossing at the *R* point, determine the topological properties of these compounds. As shown in Ref. [3], they are determined by the crystal symmetry of the noncentrosymmetric P2${}_{1}$3 (#198) space group, taking into account that both of these points are time-reversal-invariant. Topological charges at the Γ and *R* points are −4 and 4, respectively, as was calculated for RhSi [5] and for CoSi [4,6]. The linearized k–p Hamiltonian in the vicinity of the *R* point was constructed in Ref. [3], and near the Γ point in Ref. [6]. Analysis of the low-energy band structure near these nodes [6] confirmed that large topological charges are connected in the P2${}_{1}$3 group materials with a large degree of degeneracy, in contrast to SrSi${}_{2}$, where a topological charge of 2 is related to the nonlinearity of the spectrum [81]. The magnitude of topological charges implies the appearance of four unusually extended surface Fermi arcs that connect the projections of Γ and *R* points on the surface Brillouin zone [4,5,6]. For CoSi and RhSi, these Fermi arcs are plotted in Figure 6 and Figure 7.

**Figure 8:**
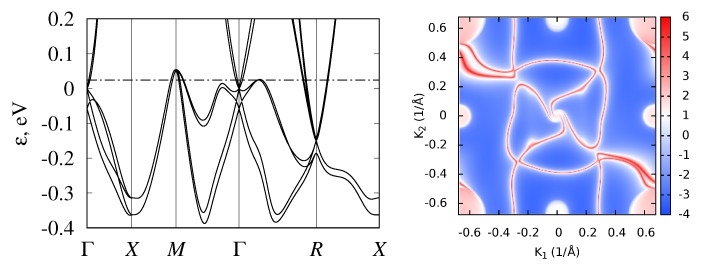
Band structure and surface Fermi arcs in CoGe.

**Figure 9:**
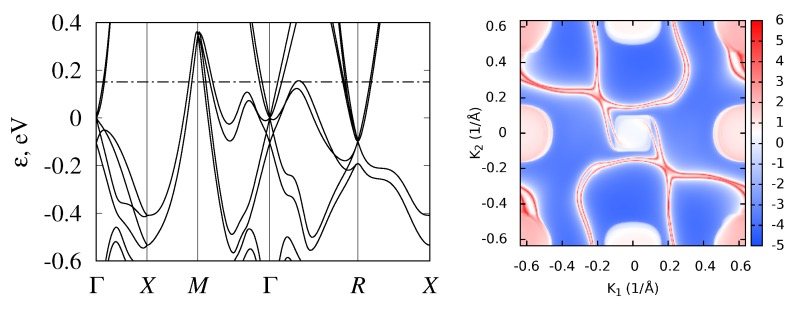
Band structure and surface Fermi arcs in RhGe.

**Figure 10:**
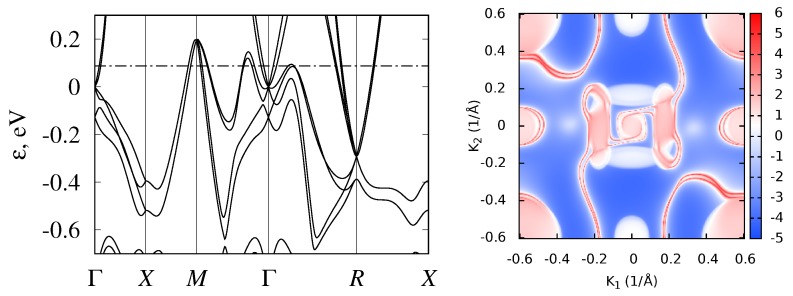
Band structure and surface Fermi arcs in RhSn.

Isostructural compounds CoGe, RhGe, and RhSn are also semimetallic. Their band structures are shown in Figure 8, Figure 9 and Figure 10. Calculations showed that, in these monosilicides, band crossings at the Γ and *R* points have the same topological charge (±4), and surface states also demonstrated long Fermi arcs. The valence-band maxima at *M* point and on Γ−R and Γ−M lines are higher in energy than the Fermi level, so Fermi arcs have more complex shapes compared to those in CoSi and RhSi. To compare their band structures, some inter-band energy gaps are given in Table 3 .

Without spin–orbit coupling, there is a single threefold degenerate (without accounting for spin) node at the Γ point, with wave functions transforming under symmetry operations of a little space group according to three-dimensional single-valued representation $\Gamma_{4}$, using notations from Ref. [82]. In Refs. [3,4], low-energy excitations at this node are called “spin-1 fermions”. Without SOC, the topological charge of this node is equal to −2.

Spin–orbit coupling splits this node into four- and twofold degenerate ones, separated in energy by $\Delta_{\Gamma }$. Wave functions of the fourfold degenerate node are transformed following the direct sum of two two-dimensional mutually conjugated double-valued representations ${\bar{\Gamma }}_{6}+{\bar{\Gamma }}_{7}$, paired as a result of time-reversal symmetry. The total topological charge of this node is −4. Split-off twofold degenerate simple Weyl node has topological charge 1. Its two wave functions are transformed according to two-dimensional double-valued pseudoreal representation ${\bar{\Gamma }}_{5}$, for which time-reversal symmetry does not increase degeneracy. In CoSi and CoGe, spin–orbit coupling is smaller than in RhSi, RhGe, and RhSn, thus this splitting is about $\Delta_{\Gamma }\approx$ 50 meV in Co-based semimetals, while it is almost twice larger in Rh-based ones (see Table 3).

The node at the *R* point in all considered semimetals was lower than the node at the Γ point by $\Delta_{\Gamma R}$, which varied from 0.1 eV in RhGe to about 0.5 eV in RhSi. At the *R* point without SOC, there is a fourfold degenerate node (without accounting for spin degeneration), called in Ref. [15] “charge-2 fermions”. Its topological charge without SOC is 2. Four wave functions at this point transform according to the sum of a pair of two-dimensional mutually conjugated single-valued complex representations $R_{1}+R_{3}$, combined due to time-reversal symmetry.

Due to spin–orbit interaction, the node at the *R* point splits into six- and twofold degenerated ones, $\left(R_{1}+R_{3}\right)\times D_{1/2}\to \left({\bar{R}}_{7}+{\bar{R}}_{7}\right)+\left({\bar{R}}_{5}+{\bar{R}}_{6}\right)$, where $D_{1/2}$ is the spinor representation. Wave functions of a sixfold degenerated level transform according to two real three-dimensional double-valued representations ${\bar{R}}_{7}$, doubled because of time-reversal symmetry. Mutually conjugated one-dimensional double-valued complex representations ${\bar{R}}_{5}$ and ${\bar{R}}_{6}$ form a two-dimensional representation of the split-off level. Spin–orbital splitting varies from $\Delta_{R}\approx$ 30 to 95 meV, and is larger in Rh-based semimetals than in Co-based ones (see Table 3). The topological charge of a sixfold degenerated node at *R* point equals to 4. Because of pair-wise degeneracy of bands at the surface of Brillouin zone, non-Abelian Berry curvature should be used [6] and topological charge for pair of bands, corresponding to split-off doublet, is equal to zero.

For CoSi, the bulk electronic spectrum and Fermi arcs were observed in ARPES experiments in Refs. [14,15,16]. The experimental bulk band structure and Fermi arcs correlate quite well with the results of first-principle calculations [4,5,6]. Unfortunately, because of the small spin–orbital splitting in CoSi, it was not possible to experimentally resolve closely lying pairs of Fermi arcs, and the interpretation of the results in Refs. [14,15,16] was made without SOC.

### 3.3. Semiconducting-Monosilicide Band Structure

As mentioned in the Introduction, FeSi, RuSi, and OsSi are semiconductors. Their calculated energy gaps are given in Table 4 in comparison with the experimental data. For comparison, energy gaps were calculated both without and with SOC at the same lattice-parameter values, given in Table 2. It can be seen that splitting bands due to spin–orbit coupling leads to smaller values of calculated energy gaps $\epsilon_{g}$. This decrease in $\epsilon_{g}$ is larger for RuSi and OsSi, compared to FeSi, due to the increase of the magnitude of SOC effects.

The most studied semiconducting material from the family of transition-metal monosilicides is FeSi. Our calculated value of $\epsilon_{g}$ is similar to results from [23,83]. Theoretical $\epsilon_{g}$ is approximately twice larger than experimental values 0.054–0.06 eV [27,28,29]. As is apparent from the following discussion, this overestimation is connected with the effects of dynamical electronic correlations.

Density functional theory in GGA approximation commonly underestimates the band gap of semiconductors due to many-body effects. Usually, this underestimation can be corrected by using hybrid functionals, better describing nonlocal exchange effects in electron–electron interactions. For FeSi, a B3LYP hybrid functional and Hartree–Fock calculations were performed in Ref. [84]. As expected, they gave larger band gaps compared to the PBE calculations: 1.531 and 3.362 eV, respectively [84]. Our calculations using a HSE03 hybrid functional also led to a similar result, $\epsilon_{g}=1.13$ eV. Thus, using a hybrid functional in this case did not lead to any improvement in band-gap description.

There were also attempts to take into account local electron–electron correlations using the Hubbard model in static (LDA+U) or dynamical mean-field theory (DMFT) approaches. Required values of parameters *U* and *J*, describing on-site Coulomb and the exchange interactions of *d*-electrons can be taken from a comparison with the experiment [85] or obtained from GW [86] or constrained DFT [87] calculations. In general, these values vary in the literature in quite a broad range. For example, in Ref. [88], the LDA+U approach was used for FeSi to predict a nonmagnetic insulator to ferromagnetic metal transition in a magnetic field for Hubbard parameter U> 3.2 eV. The DMFT approach was used for FeSi and its alloys with CoSi in Ref. [85], with the value of effective Hubbard parameter being $U_{eff}=U-J=$ 1 eV, for FeSi in Ref. [86] with U= 5.0 eV and J= 0.7 eV, and for FeSi and CoSi in Ref. [87] with U= 4.4 eV and J= 0.82 eV. Our calculations in the framework of the simplified rotationally invariant LDA+U approach [89] gave a band gap of $\epsilon_{g}$ = 0.16 eV for $U_{eff}=$1 eV and 0.76 eV for $U_{eff}=$ 4.3 eV, thus not leading to improved $\epsilon_{g}$ values compared to the GGA approximation.

On the other hand, dynamic electron correlations in Refs. [85,86,87] predicted a decrease of the band gap to approximately 0.05 eV at low temperatures. In agreement with the experiment, these calculations showed further decrease of the band gap with the rise of temperature. According to Mazurenko et al. [85], the band gap disappeared at 386 K. These results suggest that, in FeSi, dynamic on-site correlations of Fe d-electrons are important for correct band structure description.

Interesting results concerning the broadening of the dispersion lines due to the finite lifetime of coherent states connected with on-site correlations were obtained in Ref. [87]. In the case of FeSi, the degree of incoherence increases with temperature, and the band gap decreases from 100 to 500 K, and then increases above 500 to 800 K. In contrast to FeSi, the degree of band-state incoherence in CoSi is small, in the range of ±0.3 eV near the Fermi level, and decreases with the temperature [87]. Thus, for CoSi, DFT seems to give quite accurate results. This conclusion was indirectly confirmed by the better agreement of the calculated lattice constants and bulk moduli for CoSi compared to other monosilicides of the fourth period.

**Figure 11:**
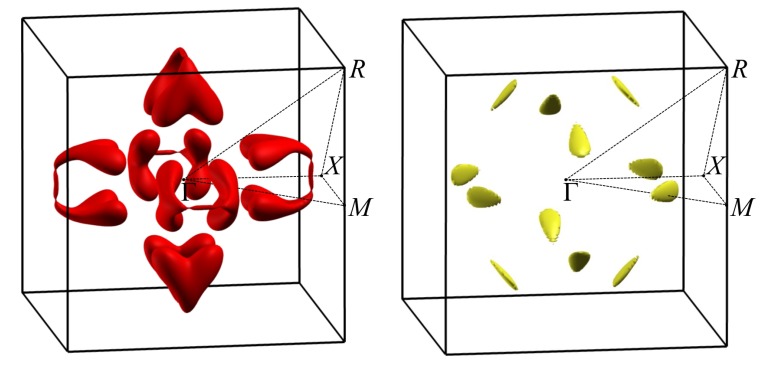
Constant-energy surfaces of valence (**left**) and conduction bands (**right**) of FeSi at energies of VBM and CBM. Energies of VBM and CBM are shown in Figure 2 by dotted lines.

Another interesting feature of the FeSi band structure is the complex constant-energy surfaces, as was noted in Ref. [83], where the bands structures of FeSi, RuSi and OsSi were calculated without SOC. Our calculations without SOC agree with the $\epsilon_{g}$ values obtained in Ref. [83]. The authors of Ref. [83] noted that the position of the conduction band minimum (CBM) and the valence band maximum (VBM) actually did not lie on high-symmetry lines, and that the constant-energy surfaces are strongly nonellipsoidal. Our calculations with SOC inclusion confirm this conclusion. Therefore, band gaps determined for FeSi from the energy spectrum, given in Figure 2, are about 6 meV larger than the band gap obtained from the density of states, calculated on the fine grid using tetrahedral interpolation. At the same time, for RuSi and OsSi, this difference was almost negligible (<1 meV). Although this difference is small even for FeSi, it leads to the strong energy dependence of density of states near the band edges. The density of states for FeSi, RuSi, and OsSi near the band gap are shown in Figure 5. It can be seen that, passing from FeSi to OsSi, DOS sharpness at the band edges decreases. To better illustrate this, in Figure 11, we plotted constant-energy surfaces for FeSi, calculated for energies at the band edges, shown by dashed lines on the band diagram (Figure 2). In Figure 11, it can be seen that these energy surfaces have nonzero volume with a very unusual shape due to the location of the band extrema, away from high-symmetry lines in the Brillouin zone.

In the case of FeSi, $\epsilon_{g}$ values, calculated without and with SOC, are larger than those obtained in experiments; on the contrary, for RuSi and OsSi, they are smaller than the experimental values. Although calculations using a HSE03 hybrid functional lead to an increase of $\epsilon_{g}$ from 0.146 to 0.47 eV for RuSi and from 0.18 to 0.53 eV for OsSi, these values appeared to be larger than the experimental ones.

### 3.4. Band Structure of Metallic Monosilicides

In CrSi, MnSi, TcSi, and ReSi, the Fermi level is shifted downward compared to eighth-group semiconducting monosilicides; thus, they demonstrate metallic behavior (Figure 2, Figure 3 and Figure 4). Solid solutions of the metallic and semiconducting monosilicides can be used to tune the Fermi level to a desired position. All these materials demonstrated numerous two- and fourfold degenerate band crossings at Γ point, and a sixfold band crossing at *R* point. The formation of ReSi–OsSi solid solutions, for example, can be used to shift the Fermi level close to a fourfold band crossing at Γ point. In these two compounds, a band crossing at the *M* point can also be clearly seen. At the same time, there is large background DOS associated with other branches of the electronic spectrum at energies close to these nodes. Therefore, it would probably be difficult to experimentally detect these nodes.

## 4. Influence of Band Structure Peculiarities on Monosilicide Thermoelectric Properties

Electronic band structure determines not only topological but also, to a large extent, transport properties. Many considered monosilicides were studied as thermoelectric materials. They can be divided into three classes. In the semimetallic monosilicides of Co and Rh, there is no energy gap near the Fermi level, but the density of states at $\epsilon_{F}$ is small. Fe, Ru, and Os monosilicides are semiconductors. In Cr, Mn, Tc, and Re monosilicides, the Fermi level is situated inside the valence band with a high density of states, and their transport behavior is similar to that of metals.

**Figure 12:**
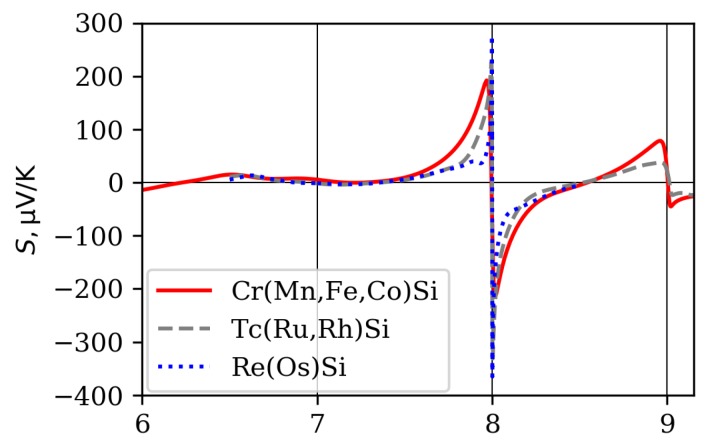
Room temperature Seebeck coefficients of considered transition-metal monosilicides of groups 6–9 of the Periodic Table, and of quasibinary alloys of intermediate compositions.

For the CrSi–MnSi–FeSi–CoSi–Co${}_{0.85}$Ni${}_{0.15}$Si alloy series, calculations based on an ab initio band structure, using rigid-band and constant relaxation time approximations (CRTA), were performed in Ref. [10]. Our calculations for the fourth-period monosilicides are shown in Figure 12 and agree with the results of Ref. [10]. For comparison, we made similar calculations for the TcSi–RuSi–RhSi and ReSi–OsSi series of compounds. These curves qualitatively reproduce experimental dependence of the Seebeck coefficient on the composition: in metallic monosilicides, thermopower is small; in semiconducting ones, it is strongly dependent on doping, including sign change. The increase of Seebeck coefficient amplitude in semiconducting monosilicides is in accordance with the increase of the energy gap while passing from FeSi to RuSi and OsSi. Qualitatively, these dependences fit into the common theory of the Seebeck coefficient.

However, to improve agreement with the experiment, one has to consider real scattering mechanisms beyond CRTA. This effect can be considered, using CoSi or GeSi semimetals as an example. In these materials, the electronic spectrum near the Fermi level is quite complicated and contains both electron- and hole-like bands, and also branches with parabolic-like and nearly linear dispersion.

In Ref. [2], attention was drawn to a change of the dispersion relation and the dependence of the density of states on energy in Weyl semimetals, compared to materials with a conventional parabolic dispersion law. Instead of square-root dependence of the density of states on energy $g_{p}∼\epsilon^{1/2}$, the density of states in Weyl or Dirac semimetals increases quadratically with energy $g_{w}∼\epsilon^{2}$. In relaxation-time approximation with $\tau ∼\epsilon^{r}$, the usual expression for Seebeck coefficient for the case of linear dispersion reads [2]:

$$S=\pm \frac{k_{B}}{e}\left(\frac{\int_{0}^{\infty }\left(-\partial f_{0}/\partial x\right)\;x^{r+3}dx}{\int_{0}^{\infty }\left(-\partial f_{0}/\partial x\right)\;x^{r+2}dx}-y\right),$$

where $f_{0}$ is the Fermi distribution function, and *x* and *y* are energy and chemical potential in $k_{B}T$ units. In materials with parabolic dispersion, exponents in integrands should be replaced with r+5/2 and r+3/2, correspondingly. The faster the integrand function increases (larger *r*), the greater the relative contribution of high-energy carriers is, and the larger the Seebeck coefficient is. Hence, in constant relaxation-time approximation (r=0), the Seebeck coefficient should be larger in the case of linear, compared to parabolic dispersion. On the contrary, if relaxation time is inversely proportional to the density of states (r=−1/2 for parabolic dispersion and r=−2 for linear one), the situation changes and the Seebeck coefficient is larger for the case of the parabolic band. This simple example illustrates that the value of the Seebeck coefficient substantially depends on both dispersion law and scattering mechanism.

In CoSi and its dilute alloys with FeSi and NiSi, the Seebeck coefficient calculated in CRTA approximation deviates from the experiment, both in absolute value and in composition corresponding to the |S| maximum. The largest experimental value of −80μV/K at 300 K was reached for stoichiometric CoSi composition [7], while CRTA approximation predicted a value of −45μV/K, which was reached at 1% of Ni doping. Deviation could be caused by both the change of electronic band structure in alloys, and by energy dependence of relaxation time. For example, for stoichiometric CoSi, contribution to the Seebeck coefficient from heavy hole-like bands in constant relaxation time approximation prevail, leading to a positive Seebeck coefficient in contrast to experiment. In CoSi and its solid solutions with a small addition of Ni or Fe, the magnitude of Seebeck coefficient can be quite well described taking into account electron–phonon interactions and point-defect scattering [90]. The energy dependences of these scattering rates, obtained by means of first-principle calculations, appeared to be proportional to the total density of states, implying the importance of interband scattering. In CoSi, the Fermi level is situated just below the node at the Γ point ($\epsilon_{\Gamma }=0$) near the minimum of the density of states (the energy dependence of DOS for CoSi is similar to that of FeSi, which is plotted in Figure 5a). A stronger increase and larger DOS values below the Fermi level, compared to the above $\epsilon_{F}$, lead to stronger scattering of charge carriers below the Fermi level and results in a large negative Seebeck coefficient, in agreement with the experiment. For the same reason, resistivity of Fe-doped CoSi samples becomes larger compared to Ni-doped ones. Therefore, in the case of semimetallic monosilicides, band structure is important for the description of transport properties both directly and for describing the energy dependence of the relaxation time, since CRTA is not a good approximation in this case.

## 5. Summary

The comparison of electronic band structures of transition-metal monosilicides of B20 (FeSi-type) crystal structure was performed on the basis of unified first-principle calculations supplemented by review of available theoretical and experimental data. We included our results for the band structures of TcSi, ReSi, and RhSn in generalized gradient approximation with accounting for spin–orbit coupling, which we did not find in the literature. We discussed the influence of the band structure on their topological and thermoelectric properties.

From this point of view, the most interesting monosilicides are semimetallic CoSi and RhSi. The symmetry of a B20 (FeSi-type) crystal structure determines the existence of four- and sixfold band crossings at the Γ and *R* points of the Brillouin zone. In CoSi and RhSi, the Fermi level is close to these band crossings and, at the same time, it is close to the minimum of the density of states. Thus, their topological properties should not be strongly obscured by contributions from other bands. For example, they demonstrate well distinguishable, elongated surface Fermi arcs, connecting projections of Γ and *R* points on the surface Brillouin zone. Isostructural compounds of CoGe, RhGe, and RhSn are also semimetallic and demonstrated elongated surface Fermi arcs, but their form is more complex because of the contribution of background density of states around the Fermi level.

The thermoelectric properties of CoSi are also interesting because it has a Seebeck coefficient that is large for semimetals, which cannot be described in constant relaxation-time approximation. In semimetals, the Seebeck coefficient is determined by the balance of electron and hole contributions. In CoSi, the density of states determines this balance both directly and indirectly through strong non-symmetric energy dependence of scattering rate, which is proportional to DOS.

Fe, Ru, and Os monosilicides are semiconductors. They can have large thermopower strongly dependent on doping and they are topologically trivial insulators.

Mn, Cr, Tc, and Re monosilicides are metals. Their thermopower is small. They also posses linear band crossings at Γ and *R* points. The Fermi level can be positioned close to these points, e.g., by means of alloying. In these materials, topological properties are more difficult to detect because of large background density of states at the intermediate points of the Brillouin zone.

## Figures and Tables

**Table 1:** Formation ($\epsilon_{\mathrm{form}}$) and decomposition ($\epsilon_{\mathrm{hull}}$) energies following density functional theory (DFT) calculations [65].

Material	$\epsilon_{\mathrm{form}}$, eV/at	$\epsilon_{\mathrm{hull}}$, eV/at	Decomposes into
CrSi	−0.296	0.069	Cr${}_{3}$Si(Pm$\bar{3}$n) + CrSi${}_{2}$(I4/mmm)
NiSi	−0.401	0.101	NiSi(Pnma)
RhSi	−0.787	0.032	RhSi(Pnma)
OsSi	−0.367	0.012	Si${}_{3}$Os${}_{2}$(Pbcn) + Os(P6${}_{3}$/mmc)
IrSi	-	-	IrSi(Pnma)
CoGe	−0.210	0.006	CoGe(C2/m)
CoSn	-	-	CoSn(P6/mmm)
RhGe	−0.507	0.040	RhGe(Pnma)

**Table 2:** Lattice parameters and bulk moduli for B20 monosilicides of different transition metals, obtained by fitting to the Birch–Murnagan equation of state, including atomic-force relaxation for zero magnetic moment and allowing for nonzero magnetic moment (in parentheses). Experimental values were taken for comparison from Refs. [31,66,68,69,70,71,72,73,74,75,76,77,78,79,80].

Material	$a_{0}$, Å	$x_{\mathrm{Me}}$	$x_{\mathrm{Si}}$	$B_{0}$, GPa
CrSi (0.48 $\mu_{B}$/at)	4.5895 (4.5947)	0.1377 (0.1379)	0.8477 (0.8473)	207 (196)
	4.607 [69], 4.629 [70]			
MnSi (1 $\mu_{B}$/at)	4.4974 (4.5180)	0.1374 (0.1366)	0.8456 (0.8452)	221 (210)
	4.5598 [68], 4.557 [71]	0.138 [71]	0.846 [71]	164 [68]
FeSi	4.4489	0.1363	0.8408,	219
	4.483 [68], 4.489 [72]	0.137 [72]	0.842 [72]	186 [68]
CoSi	4.4302	0.1451	0.8432	219
	4.444 [68], 4.4445 [73]	0.144 [73]	0.846 [73]	207 [68]
TcSi	4.7722	0.1348	0.8448	229
	4.755 [74]			
RuSi	4.7361	0.1295	0.8376	209
	4.7058 [31], 4.7059 [75]	0.1365 [31], 0.1302 [75]	0.8426 [31], 0.8387 [75]	
RhSi	4.7199	0.1523	0.8419	222
	4.6750 [76], 4.6740 [77]	0.1440 [76], 0.1459 [77]	0.8400 [76], 0.8403 [77]	
ReSi	4.8021	0.1343	0.8443	248
	4.7744 [78]	0.1346 [78]	0.8375 [78]	
OsSi	4.7927	0.1234	0.8357	209
	4.7290 [79]			
CoGe	4.6424	0.1360	0.8394	155
	4.637 [66]			
RhGe	4.9405	0.1211	0.8351	98
	4.862 [66]			
RhSn	5.1986	0.1430	0.8397	141
	5.122 [80]			

**Table 3:** Evolution of energy differences for semimetallic monosilicides and monogermanides of Co and Rh, and for RhSn crystallizing in B20 structure. $\Delta_{\Gamma }$ is SOC splitting of two- and fourfold degenerate nodes at Γ point. $\Delta_{R}$ is the SOC splitting of the two- and sixfold degenerate nodes at *R* point. $\Delta_{\Gamma R}$ is energy distance between sixfold degenerate node at *R* point and fourfold degenerate node at Γ point. $\Delta_{\Gamma M}$ is energy difference between valence band maximum at *M* point and fourfold degenerate node at Γ point.

Material	$\Delta_{\Gamma }$, eV	$\Delta_{R}$, eV	$\Delta_{\Gamma R}$, eV	$\Delta_{\Gamma M}$, eV
CoSi	−0.053	−0.031	−0.225	−0.049
RhSi	−0.111	−0.060	−0.511	−0.082
CoGe	−0.058	−0.038	−0.148	0.053
RhGe	−0.106	−0.095	−0.098	0.347
RhSn	−0.128	−0.097	−0.291	0.194

**Table 4:** Energy gaps in semiconducting monosilicides of Fe, Ru, and Os crystallizing in B20 structure.

Material	$\epsilon_{g}$ (SOC), eV	$\epsilon_{g}$ (without SOC), eV	$\epsilon_{g}$ (expt.), eV
FeSi	0.111	0.196, 0.17 [83], 0.13 [23]	0.054 [29], 0.055 [27], 0.06 [28]
RuSi	0.146	0.235, 0.22 [83], 0.48 [24], 0.22 [23]	0.26 [31], 0.2–0.3 [30]
OsSi	0.182	0.518, 0.74 [24], 0.68 [26], 0.53 [23], 0.50 [83]	>0.26 [31]
